# Brenda Linares, MLIS, MBA, AHIP, Medical Library Association President, 2024-2025

**DOI:** 10.5195/jmla.2026.2449

**Published:** 2026-04-01

**Authors:** Annabelle V. Nuñez, Kate Flewelling, Emily Vardell, Kristine M. Alpi

**Affiliations:** 1 Director, Health Sciences Library, University of Arizona Health Sciences Library, Tucson, AZ; 2 Director, Library Services, Medical Campus, Boston University, Boston, MA; 3 Associate Professor, School of Library and Information Management, Emporia State University, Emporia, KS; 4 Associate Dean of Libraries and Information Sciences, Scholarly & Research Technologies, Icahn School of Medicine at Mount Sinai, New York, NY

**Keywords:** Biography, MLA President, Latina, Latinx

## Abstract

In this profile, Brenda M. Linares, AHIP, Medical Library Association (MLA) president 2024-25 is introduced through a discussion of her contributions and commitment to growing the next generation of Latina library leaders. As the first Latina immigrant MLA president, she partnered with colleagues to build organizational structures to strengthen diversity, equity, and inclusion in multiple regional chapters of MLA. In addition to her focus on integrating her family into her professional engagements, Linares brought a strong business orientation from her bachelor's degree in Finance and her Master of Business Administration to her MLA leadership and to her professional role as Associate Dean of Library Services, University of Missouri-Kansas City (UKMC) Libraries.

Brenda Marisol Linares was born on Christmas Day 1978 in Jutiapa, Guatemala. She immigrated to the United States at the age of ten and grew up in the San Fernando Valley, California, with her parents Rogelio and Leticia and her sister Iveth. As a non-native English speaker, she faced barriers when beginning school in the United States, but her 5th grade teacher Dr. Deborah Neal (with whom she is still in touch!) recognized her intelligence and hard work. Dr. Neal helped Brenda apply to the Magnet program in her school. This early recognition of her talents is one example of the mentors in Brenda’s life who have supported her in achieving her dreams and her path to success.

## BRENDA LINARES’ PROFESSIONAL JOURNEY

Brenda’s first introduction to working in libraries was as a student library assistant at California State University, Northridge (CSUN) while pursuing her undergraduate degree in finance. Through this work, she met Dr. Karin Durán, Head of the Teacher Curriculum Center (TCC) in the CSUN University Library. After graduation, Brenda was considering meaningful career pathways when Karin encouraged her to consider pursuing her Master of Library Science. Karin’s example and mentorship paved the way for Brenda’s passion for working in librarianship.

Brenda completed her Master of Library & Information Science in the UCLA School of Education & Information Studies. In her final semester as an MLIS student, Brenda worked as a graduate library assistant in four separate university libraries, including a semester-long internship at UCLA’s Louise M. Darling Biomedical Library, where her interest in medical librarianship blossomed.

After graduating in 2007, Brenda began her career in the competitive National Library of Medicine (NLM) Associate Fellowship program, which changed her life professionally and personally. In the first year of the fellowship, she learned all aspects of NLM and conducted independent projects, including an Assessment of the Central American Network for Disaster and Health Information (CANDHI). It was in the NLM Associate Fellowship that she met her future wife, Emily Vardell. Their career paths grew together over the years through several moves.

In 2008, Brenda moved to the University of Miami for the second year of the fellowship. Her acumen in outreach, instruction, and operations led to her being hired as Administrative Librarian and Manager, Finance. She was later promoted to Head of Library Outreach and Community Engagement, in recognition of her work as project manager of the library’s National Network of Libraries of Medicine (NNLM)-funded outreach project in the Department of Community Service engaging in health fairs across south Florida communities [1]. After working at the University of Miami, Brenda and Emily moved to Chapel Hill, North Carolina, where Brenda worked as Librarian and Outreach Liaison at the Health Sciences Library at the University of North Carolina at Chapel Hill, liaising to the Institute on Aging, providing outreach services to Area Health Education Centers (AHEC), and supervising library and information science graduate student field experiences. Committed to lifelong learning, she simultaneously pursued her Master of Business Administration (MBA) through the Working Professional Online MBA program at the Jenkins School of Business at North Carolina State University.

In 2017, Brenda and Emily moved to Kansas, where Brenda began her position as Research and Learning Health Sciences Librarian at the A.R. Dykes Library at the University of Kansas Medical Center. As liaison to the School of Nursing, she brought her expertise and passion for outreach to underserved populations by spearheading an NNLM funded project that developed health-related podcasts for the Latinx community in Kansas City [2,3]. In 2022, she was tapped for the role of Associate Dean of Library Services at the University of Missouri-Kansas City UMKC Libraries, where she supervises seven professional librarians and oversees six library departments, including the UMKC Health Sciences Library.

## LEARNING ALONGSIDE AND LEADING ASSOCIATION COLLEAGUES

Brenda joined the Medical Library Association (MLA) in 2008 and advanced to Distinguished Membership in its Academy of Health Information Professionals (AHIP) in 2019. Brenda is passionate about continually learning new things, works with her fellow librarians to share knowledge, and is a terrific teammate. She applies her outreach librarian skills frequently, helping ensure that MLA members and other librarians have collaborators and do not feel alone and isolated. She looks to her community of practice to contribute her expertise and grow her skills through volunteering on projects that advance our profession. Brenda is persistent and encourages others to persevere. She had volunteered to work on one of the MLA Research Agenda systematic reviews on the topic of librarian impact on informatics education for health professions. In 2014, when many people had to step away from the project, Brenda remained committed and alternated leadership with the first author in scheduling meetings and keeping us together which ultimately led to the paper being published in 2021 [4].

Since Brenda’s career began in 2007, she has led the profession in expanding opportunities for all members through multiple diversity, equity, and inclusion (DEI) programs. Two years before MLA created a Diversity, Equity and Inclusion Task Force (now the Community Building and Belonging Committee [5]), Brenda served as the inaugural chair of the MidAtlantic Chapter (MAC) Diversity Task Group [6]. In 2018, MAC received the MLA Chapter of the Year award for the Task Group’s efforts, and, in 2020, the Task Group received the MAC Marguerite Abel Service Recognition Award. By then, Brenda had moved to the Midcontinental Chapter of the Medical Library Association where she was the inaugural chair of that chapter’s Diversity and Inclusion (D&I) Task Force [7]. In 2021, Brenda was awarded the MLA President’s Award for efforts on the MLA Spanish-Language COVID-19 Resources Page. Her expertise developing chapter level DEI task forces led to her involvement in supporting the creation of the third DEI-related committee she has helped establish - the JEDI (Justice, Equity, Diversity, and Inclusivity) Committee in the South Central Chapter of MLA.

Brenda’s commitment to diversity, equity, and inclusion has never been something she “adds on” to her work; it’s the throughline. Long before DEI initiatives gained broader momentum, she was already working to break down barriers to health information access, asking who gets left out and who is expected to navigate complex health systems as an outsider.

When beginning her path to librarianship in 2005, Brenda was selected to participate in the American Library Association (ALA) Spectrum Scholars Program. This two-year program is geared toward library students and is foundational in preparing library and information professionals. It is where she found both community and purpose. That experience grew out of her deeply rooted belief that representation and opportunity are not abstract values, but critical to the infrastructure of an equitable profession. Soon after, it was both fortuitous and fitting that she was selected for the NLM Associate Fellowship Program, intentionally designed to cultivate future leaders from diverse educational and cultural backgrounds. The fellowship didn’t just sharpen her skills; it expanded her platform. Brenda continued her relationship with ALA after time as a scholar and became a presenter, returning repeatedly to share what she had learned, open doors, and build real connections with new and emerging librarians who needed to see themselves in the profession.

By 2012, recognizing that MLA had few visible representatives from the Latinx community, Brenda’s advocacy took on a collective form. She worked tirelessly alongside Diana Almader-Douglas and Annabelle Nuñez to establish the MLA Latinx Special Interest Group (now Caucus) in 2014. The aim was to create space for building a network when isolation was common and to ensure that Latinx voices could gather, lead, and be heard. That work was more than organizational; it was an act of embracing belonging, and it continues to ripple outward in MLA, as evidenced in the continuation of this group. In 2021, the collective momentum helped inspire members of the caucus to publish the JMLA commentary, “¡Presente: Affirming Latinx voices with health sciences library scholarship”, which outlines a call to action to “empower Latinx and BIPOC to have their voices heard, supported, and cultivated.” [8]

Brenda’s leadership is marked by progression and continuity; she has been involved in different capacities within MLA with committees, national program committees (NPCs), juries, and other leadership roles. While wrapping up her term as Past-Chair of the Leadership and Management Caucus (then Section), she transitioned immediately to being elected to the Board of Directors starting May 2020. Whenever she joins a new community, she volunteers for service leader roles at the state and regional level. In the Association of North Carolina Health and Sciences Libraries (ANCHASL), she chaired the Membership Committee and then progressed to Secretary before moving to Kansas and then becoming active in Midcontinental Chapter activities, where she was recognized in 2023 with the Bernice M. Hetzner Award for Excellence in Academic Health Science Librarianship. Embracing state and regional leadership roles, as well as championing leading from the middle are hallmarks of Brenda’s contributions.

Brenda’s approach to leadership has also been profoundly shaped by an unwavering belief that health information should never be a privilege reserved for the few. This guiding principle became a consistent thread in her guest lectures across multiple Library and Information Science (LIS) courses at the University of North Carolina’s School of Information and Library Science and Emporia State University’s School of Library and Information Management, as well as in her outreach and leadership activities as she has moved all across the United States from California to the east coast (Washington DC, Florida, and North Carolina) and then settling in Kansas.

## REPRESENTING HEALTH SCIENCES LIBRARIANS AT THE HIGHEST LEVEL

Kristine Alpi, MLA President from 2021-22 and chair of the Nominating Committee that invited Brenda to the MLA Presidential election slate, recalled thinking that it was not the right time, that Brenda needed to take a break after coming off an intense time on the Board of Directors with many challenging issues. There was also Brenda’s new job and new daughter, Clara, to be considered. Still when invited to be a candidate, Brenda was courageous and wanted to make the most of the opportunity. One of her special gifts is figuring out how to bring all the facets of her life together. She transformed a “not ideal” time into the right time to lead.

Brenda has always integrated her family life into her work life in a way that inspires and lowers the barrier for others who struggle to balance both. Kate Flewelling recalls meeting Brenda, her parents and sister in tow, the day before they both started their Associate Fellowship at NLM. The next day, Brenda met fellow Associate and her future wife, Emily Vardell. Over the years, Brenda and Emily have supported each other’s careers, served as co-authors on multiple publications, and grown their family to include two daughters [9] (Vardell & Linares, 2025). Their older daughter Clara regularly attends MLA conferences. One of Kate’s favorite memories is watching three-year-old Clara watching her Mamá on stage as President, awe in her eyes and wearing a “First Daughter” t-shirt. Just as they were on Brenda’s first day at NLM, Brenda’s parents and sister were in the audience.
